# Occurrence and adverse effect on outcome of hyperlactatemia in the critically ill

**DOI:** 10.1186/cc7918

**Published:** 2009-06-12

**Authors:** Houman Khosravani, Reza Shahpori, H Thomas Stelfox, Andrew W Kirkpatrick, Kevin B Laupland

**Affiliations:** 1 Department of Critical Care Medicine, University of Calgary, 1403 29th Street NW, Calgary, Alberta, T2N 2T9, Canada; 2 Department of Medicine, University of Calgary, 1403 29th Street NW, Calgary, Alberta, T2N 2T9, Canada; 3 Department of Community Health Sciences, University of Calgary, 1403 29th Street NW, Calgary, Alberta, T2N 2T9, Canada; 4 Department of Surgery, University of Calgary, 1403 29th Street NW, Calgary, Alberta, T2N 2T9, Canada

## Abstract

**Introduction:**

Hyperlactatemia is frequent in critically ill patients and is often used as a marker of adverse outcome. However, studies to date have focused on selected intensive care unit (ICU) populations. We sought to determine the occurrence and relation of hyperlactatemia with ICU mortality in all patients admitted to four ICUs in a large regional critical care system.

**Methods:**

All adults ([greater than or equal to] 18 years) admitted to ICUs in the Calgary Health Region (population 1.2 million) during 2003 to 2006 were included retrospectively. Lactate determinations were at the discretion of the attending service and hyperlactatemia was defined by a lactate level > 2 mmol/L.

**Results:**

A total of 13,932 ICU admissions occurred among 11,581 patients. The median age was 63 years (37% female), the mean APACHE II score was 25 ± 9 (n = 13,922). At presentation (within first day of admission), 12,246 patients had at least one lactate determination and the median peak lactate was 1.8 (IQR 1.2 to 2.9) mmol/L. The cumulative incidence of at least one documented episode of hyperlactatemia was 5578/13,932 (40%); 5058 (36%) patients had hyperlactatemia at presentation, and a further 520 (4%) developed hyperlactatemia subsequently. The incidence of hyperlactatemia varied significantly by major admitting diagnostic category (*P *< 0.001) and was highest among neuro/trauma patients 1053/2328 (45%), followed by medical 2047/4935 (41%), other surgical 900/2274 (40%), and cardiac surgical 1578/4395 (36%). Among a cohort of 9107 first admissions with ICU stay of at least one day, both hyperlactatemia at presentation (712/3634 (20%) vs. 289/5473 (5%); *P *< 0.001) and its later development (101/379 (27%) vs. 188/5094 (4%); *P *< 0.001) were associated with significantly increased case fatality rates as compared with patients without elevated lactate. After controlling for confounding effects in multivariable logistic regression analysis, hyperlactatemia was an independent risk factor for death.

**Conclusions:**

Hyperlactatemia is common among the critically ill and predicts risk for death.

## Introduction

Elevations of blood lactate are common in patients admitted to the intensive care unit (ICU) and have been associated with adverse outcomes [1-7]. Early studies suggested that venous lactate levels on admission to medical ICUs in patients with shock were associated with mortality rates of greater than 30% with associated correlation with the absolute lactate concentration [1-4]. More recently, both the presence of hyperlactatemia on admission and subsequent development of hyperlactatemia have been reported to be associated with greater mortality in surgical ICU patients [5-7]. Also, the rapid rate of clearance of lactate and earlier time to resolution of hyperlactatemia have been associated with increased survival probability [3,8,9].

Although it is recognized that hyperlactatemia is a common occurrence in critically ill patients and is associated with increased mortality, and that the resolution of hyperlactatemia is associated with increased survival, previous studies have focused on relatively small cohorts of selected critically ill populations [1-3,5-7,9-12]. As a result, the occurrence, determinants, and effect on outcome of hyperlactatemia in critically ill populations at large have not been well defined. The objective of the present study was to assess the incidence of hyperlactatemia and the factors associated with it in a large population of critically ill adult medical and surgical patients, and assess the effect of hyperlactatemia on mortality.

## Materials and methods

### Study population

The patients included in the study were from a regional health care system including the city of Calgary and surrounding smaller cities (population 1.2 million) [13]. Critically ill adult patients in the Calgary Health Region (CHR) are managed in closed ICUs under the care of certified intensivists in the Department of Critical Care Medicine, University of Calgary, and CHR. These include a 14-bed cardiovascular surgery ICU and three multi-system ICUs: one 24-bed multi-system ICU that serves as the regional trauma and neurosurgical referral center; one 14-bed multi-system ICU that is also the vascular surgery referral center; and a 10-bed multi-system ICU. The base study population consisted of all adults (≥ 18 years) admitted to any multi-system or the cardiovascular surgery ICU in the CHR between 1 January, 2003 and 31 December, 2006. The Conjoint Health Research Ethics Board at the University of Calgary and CHR approved this study and waived the requirement for individual written informed consent.

### Study protocol

This study utilized a retrospective cohort design. Data were obtained using the ICU Tracer database, a regional patient care, research, and administrative database that systematically and uniformly records detailed demographic, clinical, laboratory, and hospital outcome data on all patients admitted to the ICU in the CHR as previously described [14,15]. In CHR ICUs, lactate is nearly always determined from venous and arterial samples using point-of-care analysers in each of the ICUs (Radiometer ABL-725, Radiometer Copenhagen, Copenhagen, Denmark). In this study, any lactate measurement from venous or arterial blood of more than 2 mmol/L was deemed to represent hyperlactatemia. Patients that did not have a lactate measurement within the first 24 hours of ICU admission were considered to not exhibit hyperlactatemia. Severity of illness at inception (within the first day of ICU admission) was assessed using the Acute Physiology and Chronic Health Evaluation (APACHE) II score [16] and intensity of care using the Therapeutic Interventions Scoring System (TISS) [17,18].

Patients were classified into four admission categories based on available information. Cardiac surgical patients were those admitted to the cardiovascular surgery ICU. Trauma/neuro patients were those patients that were victims of major trauma as identified by the regional trauma database or had a major admission diagnosis classification listed as either trauma and/or neurologic; they could not be separated into distinct categories because of diagnostic coding limitations of the database. Other surgical patients were those admitted directly from the operating room, or those who were classified as having a surgical diagnosis as defined for the APACHE II score but excluding cardiovascular surgery, neurosurgical, and neuro/trauma patients. Medical patients were all other patients.

### Statistical analysis

Analysis was performed using Stata version 10 (Stata Corp, College Station, TX, USA). The cumulative incidence of hyperlactatemia was calculated by dividing the number of patients with at least one episode per ICU admission episode by the total number of ICU admission episodes. The average prevalence of hyperlactatemia was also calculated by dividing the number of patient-days (or part thereof) with hyperlactatemia by the total number of ICU admission days (or part thereof) and reported as hyperlactatemia-days. Although all episodes were used for cumulative incidence calculations, only each patient's first presentation to the ICU (i.e. first episode) was analyzed for evaluating mortality outcome. Mortality outcome analysis was also restricted to those patients who survived for at least one day in order to exclude those moribund at presentation.

Prior to statistical analysis the underlying distribution of all continuous variables was assessed using histograms. Normally or near-normally distributed variables were reported as means ± standard deviations and non-normally distributed variables as medians with interquartile ranges (IQR). Means were compared using the Student's t-test. Differences in proportions among categorical data were assessed using Fisher's exact test for pair-wise comparisons and the chi-squared test for multiple groups. A logistic regression model was developed to assess the independent effects of hyperlactatemia on ICU case-fatality. Variables included in the initial model were APACHE II, TISS, age, gender, admission classification, shock, and the degree of hyperlactatemia on admission. Backward step-wise variable elimination was then performed to develop the most parsimonious models.

## Results

During the three-year study period, a total of 13,932 ICU admission episodes occurred among 11,581 adult patients: 8802 (63%) were male. The median age was 63.2 (IQR = 49.7 to 73.6) years and the mean APACHE II score was 24.7 ± 8.5 (n = 13,922). Of all ICU admission episodes, 4935 (35%) were classified as medical, 4395 (32%) as cardiac surgical, 2274 (16%) as other surgical, and 2328 (17%) as trauma/neuro. Of the 8997 surgical patient admissions, 4377 (49%) were elective cases, 2690 (30%) were emergent cases, and 1854 (21%) were not immediately post-operative.

The cumulative incidence of at least one documented episode of hyperlactatemia during ICU admission was 5578/13,932 (40%), and the average prevalence was 20 hyperlactatemia-days per 100 days of ICU admission. The occurrence of hyperlactatemia varied significantly by major admitting diagnostic category (*P *< 0.001), with the highest cumulative incidence observed among neuro/trauma patients (1053/2328; 45%), followed by medical (2047/4935; 41%), other surgical (900/2274; 40%), and cardiac surgical (1578/4395; 36%). The number of hyperlactatemia-days per 100 days of ICU admission was 14.2 among neuro/trauma patients, 20.0 for medical, 20.2 for other surgical, and highest with a value of 26.1 for cardiac surgical patients (*P *< 0.001). Hyperlactatemia-days was significantly higher in patients admitted with an APACHE II score of 25 or higher (24.9. vs. 13.1; *P *< 0.001), and slightly increased in patients that were between the ages of 40 and 65 years as compared with other age ranges (21.3 as compared with 19.5 for ages 18 to 40 years, *P *= 0.003; and 19.3 for patients with age over 65 years, *P *< 0.001).

At presentation (within the first day of admission to the ICU) the median lactate concentration was 1.8 (IQR = 1.2 to 2.9) mmol/L and hyperlactatemia (lactate > 2.0 mmol/L) was identified in 5058/13,932 (36%). There was a significant relation observed between admission class and the presence of hyperlactatemia at presentation (*P *< 0.001). This was greatest in trauma/neuro patients (972/2328; 42%), followed by other surgical (814/2274; 36%), medical (1763/4935; 36%), and cardiac surgical patients (1509/4395; 34%). Of the patient admissions to the unit with hyperlactatemia (5058/13,932) documented resolution of hyperlactatemia occurred in 2994/5058 (59%) patients within a median time of 10.6 (IQR = 6.2 to 22.5) hours.

Among the 8874/13,932 patient admissions that did not have hyperlactatemia at presentation, subsequent hyperlactatemia developed in 520/8874 (6%); hyperlactatemia occurred within a median of 2.6 (IQR = 1.5 to 5.2) days after admission. The occurrence of this *de novo *hyperlactatemia in the ICU (development of hyperlactatemia among those who did not present with hyperlactatemia), was highest in medical patients (284/3172; 9%) followed by other surgical (86/1460; 6%), neuro/trauma (81/1356; 6%), and cardiac surgical (69/2886; 2%). In addition, patients with APACHE II scores of 25 or higher, were more likely to develop *de novo *hyperlactatemia (287/520; 55% *P *< 0.001). Moreover, patient age was also correlated with likelihood of developing hyperlactatemia: 10% of those aged 18 to 40 years (54/520), 36% of 40 to 65 year olds (187/520), and 54% of those aged 65 years and older (279/520; *P *< 0.001 for all). Among the 520 episodes of *de novo *hyperlactatemia, nine had documented resolution to less than 2 mmol/L within a median of 10.1 (IQR = 6.5 to 18.0) hours, while the remainder either died (153; 29%) or were discharged (358; 69%) from ICU prior to resolution.

Among the overall cohort, 1783/13,932 patients died in the ICU. The case fatality rate among the cohort of first ICU admissions for patients who survived at least the day of presentation was 1001/9107 (11%). Among the first admission cohort with at least one day of ICU stay, the fatality rate for those who presented with hyperlactatemia was significantly increased compared with those that did not present with hyperlactatemia (712/3634 (20%) vs. 289/5473 (5%); *P *< 0.001). Among patients with hyperlactatemia at presentation, the case-fatality rate was highest among medical (337/712; 47%), trauma/neuro (178/712; 25%), surgical (106/712; 15%), and cardiac surgical (91/712; 13%; *P *< 0.001). Of the *de novo *hyperlactatemia patients, when considering first admissions and a day or more of ICU stay, the case fatality was 101/379 (27%). The case-fatality rate was highest in medical (54/101; 53%) patients, then surgical (27/101; 27%), trauma/neuro (17/101; 17%), and cardiac surgical (3/101; 3%) patients.

After controlling for confounding variables in logistic regression analysis, increasing levels of hyperlactatemia at presentation were independently associated with increased risk for subsequent ICU-related mortality death as shown in Table 1.

**Table 1:** Logistic regression modeling of presenting factors associated with death in the intensive care unit

	**OR**	**95% CI**
**TISS (per point)**	1.02	1.02 to 1.03
**APACHE II (per point)**	1.08	1.07 to 1.09
**Age**	1.02	1.02 to 1.03
**Lactate concentration (max on day of admission, reference 0 to 2 mmol/L)**	1	
2 to 5 mmol/L	1.94	1.62 to 2.32
5 to 10 mmol/L	3.38	2.64 to 4.33
10 to 15 mmol/L	4.41	2.99 to 6.50
15 to 20 mmol/L	7.58	3.93 to 14.60
20-max mmol/L	10.89	4.85 to 24.48
**Admission classification medical (reference)**	1	
Cardiac surgical	0.08	0.06 to 0.11
Other surgical	0.48	0.38 to 0.60
Trauma/Neuro	1.64	1.35 to 2.01

The final model (n = 9036; among the total number of 9107 subjects data for TISS and APACHE II were only available for 9036 and 9100 cases, respectively) had good discrimination (area under the receiver operator characteristic curve = 0.852) and calibration (Hosmer-Lemeshow goodness of fit test = 1.0)

APACHE = Acute Physiology and Chronic Health Evaluation; CI = confidence interval; OR = odds ratio; TISS = Therapeutic Interventions Scoring System.

## Discussion

Our study demonstrates that hyperlactatemia is common in diverse populations of critically ill patients and that its presence at presentation or its *de novo *development during ICU stay is associated with an increased risk of death.

Previous studies have investigated the impact of hyperlactatemia on mortality in selected groups of patients admitted to the ICU and have reported cumulative incidence rates of 10 to 70% [1-7]. The observation that hyperlactatemia is associated with increased risk for death is not new [1,12]. Studies of critically ill medical [3,19,20] and surgical [9,11,21,22] patients have shown an association between elevated lactate with prolonged clearance and mortality. These rates have varied significantly partly based on the degree of mortality being related to factors such as the degree of hyperlactatemia and time to its clearance [3,9,11]. These studies have typically been limited by small size (hundreds of patients). However, no previous study has simultaneously assessed the incidence of hyperlactatemia in a large mixed cohort consisting of medical and surgical patients. Our data is important because we show that certain patient groups are at increased risk of mortality in the setting of hyperlactatemia.

Our study adds to the existing literature in the following ways. First, we directly assess medical, surgical, and neuro/trauma patients; previous studies have only looked at single populations and therefore the effect of diagnostic class is not directly comparable. Second, our findings demonstrate a stepwise relation between elevated lactate levels and an increasing degree of risk. Finally, although a number of previous studies have been powered to look at crude mortality associated with elevated lactate levels, few have been able to assess multiple confounders using logistic regression. Our study examines the largest cohort to date and had adequate power to analyze for multivariable confounders for mortality.

In several studies different cut-offs for lactate concentration were used (e.g. > 5 mmol/L) [1,2,4,5] and there appears to be a relation with increasing level and increased risk for death. Our data suggests that use of a single cut-off level for hyperlactatemia can result in loss of point-wise discrimination that appears to risk stratify the patients as observed in our cohort. It is important to note that we observed that elevated lactate at admission adds to the prediction of subsequent mortality by APACHE II score.

Although our study has several strengths related to large size and inclusion of a broad range of patients, there are a number of limitations that merit discussion. We did not assess lactate based on an algorithm but rather based on physician-dependent indications and lactate measurement was ordered as required. Therefore, we may have missed relevant cases. However, it is conceivable that the majority of such cases would be detected on routine lactate measurement as part of arterial blood gas analysis in critically ill patients. In addition, it is conceivable that in some patients hyperlactatemia was a transient event (such as with seizures), with resolution without consequence. However, such factors would bias toward a null result, and thus our findings are likely to be conservative.

We did not collect or analyze detailed clinical data and as such were not able to define the most likely clinical etiology of the hyperlactatemia. It has been suggested that the underlying cause for the hyperlactatemia, specifically the nature of the acid-base disturbance and whether a base-deficit exists may influence the predictive ability of lactate measurements [23]. In particular, the presence of a primary metabolic acidosis is highly predictive of mortality [10]. In addition, the underlying diagnosis and medical treatment can also influence the predictive capacity of lactate measurements, such as in HIV-infected patients [24,25]. Moreover, in our study most lactate measurements were obtained using the same device model of point-of-care analyzers, with the majority of samples being arterial and a small proportion being sent to a central laboratory. However, this is not likely to be a major bias as a recent study compared measurement techniques using a hand-held lactate analyzer and a bench-top blood gas analyzer to a central laboratory and found them to be in good agreement [26]. In the case of venous sampling we did not record whether they were obtained from central or peripheral sites. In quantifying hyperlactatemia-days, we assumed that where no sample was tested hyperlactemia was not present, and therefore the true rate may have been underestimated. A final limitation is that we did not look at goal-directed interventions that were employed to correct lactate levels [27].

## Conclusions

In summary, we demonstrate that hyperlactatemia is common and associated with adverse outcome. Inclusion of hyperlactatemia adds to the prediction ability of the existing APACHE II score. Moreover, we defined and compared the admission and *de novo *development of hyperlactatemia directly between different patient groups, showing that diagnostic class and absolute level of lactate elevation are important parameters. Our data suggests that there is predictive merit in routine measurement of lactate at presentation to the ICU. These findings may be useful to administrators and may allow for more accurate adjustment of mortality risk for benchmarking purposes. Similarly, clinicians can use elevated lactate levels to identify patients for potentially targeted monitoring and intervention.

## Key messages

• Hyperlactatemia occurs in approximately one-half of patients admitted to the ICU.

• Presentation with or development of hyperlactatemia after ICU admission is associated with increased mortality.

• Elevated admission lactate levels are step-wise associated with increased risk for death after controlling for severity of illness, age, and diagnosis.

## Abbreviations

APACHE: Acute Physiology and Chronic Health Evaluation; CHR: Calgary Health Region; ICU: intensive care unit; IQR: interquartile range; TISS: Therapeutic Interventions Scoring System.

## Competing interests

The authors declare that they have no competing interests.

## Authors' contributions

HK performed the primary analysis and drafted the manuscript. RS collected data and assisted with analysis. HTS and AWK contributed to study design and interpretation of data. KL conceived and designed the study, supervised the primary analysis, and assisted with manuscript drafting. All authors contributed to manuscript revision and approval.
